# Endothelial dysfunction and risk factors for atherosclerosis in psoriatic arthritis: overview and comparison with rheumatoid arthritis

**DOI:** 10.1007/s00296-024-05556-x

**Published:** 2024-03-24

**Authors:** Konrad Kaleta, Julia Krupa, Wiktoria Suchy, Anna Sopel, Mariusz Korkosz, Jarosław Nowakowski

**Affiliations:** 1 Students’ Scientific Group at the Department of Rheumatology and Immunology, Jagiellonian University Medical College, Kraków, Poland; 2 Department of Rheumatology and Immunology, Jagiellonian University Medical College, Kraków, Poland

**Keywords:** Endothelium, Atherosclerosis, Arthritis, Psoriatic, Rheumatoid

## Abstract

Endothelial dysfunction (ED) is defined as an impairment in the vasodilatory, anti-thrombotic, and anti-inflammatory properties of the cells that make up the lining of blood vessels. ED is considered a key step in the development of atherosclerotic cardiovascular disease. The association between ED and systemic inflammatory diseases is well established. However, the prevalence and clinical significance of ED in psoriatic arthritis (PsA) have been investigated to a lesser extent. This review aims to explore the link between ED and PsA, including ED in macro- and microcirculation, as well as risk factors for its occurrence in PsA and its relationship with atherosclerosis in PsA. Furthermore, the ED in PsA was compared with that of rheumatoid arthritis (RA). Regarding ED in the microcirculation, the coronary flow reserve was found to be significantly reduced in individuals with PsA. The relationship between PsA and macrovascular ED is more pronounced, along with more advanced atherosclerosis detected in patients with PsA. These results are consistent with those obtained in RA studies. On the other hand, arterial stiffness and signs of vascular remodeling were found more frequently in RA than in PsA, with the potential role of efficient anti-TNF treatment in patients with PsA and psoriasis explaining this finding. The impact of ED on cardiovascular diseases and the burden of this risk caused independently by PsA have not yet been precisely established, however, this group of patients requires special attention with regard to cardiovascular events.

## Introduction

Psoriatic arthritis (PsA) is a chronic inflammatory musculoskeletal disorder that often accompanies psoriasis (PsO). PsO affects 1–3% of the general population, and up to 30% of these individuals develop PsA [1, 2]. The American College of Rheumatology recognized PsA as a distinct clinical entity in the 1960s. Initially perceived as benign, recent findings indicate that the severity of PsA is comparable to that of rheumatoid arthritis (RA), significantly affecting quality of life and joint functionality. PsA can manifest in various forms, such as axial or peripheral arthritis, enthesitis, dactylitis, or a combination of these [3]. Approximately 90% of patients with PsA exhibit typical dermatological symptoms of PsO, although PsA can arise without skin manifestations [2]. In about 75% of cases, PsA is preceded by PsO, but in 10–15% of cases, the sequence may be reversed. The occurrence of PsA without PsO is also possible and may pose a diagnostic challenge [4]. Both RA and PsA share several features, such as chronic progression, inflammatory symptoms, and an association with increased cardiovascular (CV) morbidity and mortality, including a higher prevalence of traditional cardiovascular risk factors (CVRF) compared to controls [5]. Endothelial dysfunction (ED), a key link between RA, PsA, and CV diseases, is an early indicator of atherosclerosis [6] and plays a significant role in the progression of RA, PsA, and related comorbidities. Studies have highlighted the association of PsA with increased CV morbidity and mortality [7, 8]. Although patients with PsA often have classical CVRF such as diabetes, hypertension, hyperlipidemia, obesity, and smoking [8, 9], there are still other factors to consider in the pathogenesis of atherosclerotic cardiovascular disease (ASCVD) and, consequently, CV mortality. Despite similarities, RA and PsA differ in terms of CV risk burden and CV comorbidity profiles. These differences are also reflected in the results of studies investigating ED and signs of subclinical atherosclerosis and vascular remodeling. The impact of PsA and RA on the endothelium has been the subject of numerous studies. This article synthesizes peer-reviewed literature on the impact of PsA on endothelial function in both macrocirculation and microcirculation, comparing it with RA to provide a comprehensive perspective.

### Search strategy

Considering previously published recommendations, our strategy was to search for accessible studies focusing on ED in patients with PsA and patients with RA, published by September 1, 2023. To ensure a comprehensive and thorough data collection, we utilized a range of online databases including MEDLINE, Cochrane and EMBASE. Additionally, we employed Google Scholar as a secondary verification tool to cross-check and augment our search results. The search methodology incorporates specific commands that integrate the phrases "psoriatic arthritis" AND "endothelial dysfunction". For a more advanced search, the methodology combines the name of the disease with specific indicators that evaluate endothelial dysfunction across various levels. These indicators include measure in microcirculation—coronary flow reserve, and in macrocirculation, which encompasses assessments like flow-mediated dilatation, carotid total plaque area, coronary plaque burden, and carotid plaque burden. Additionally, the search extends to evaluating arterial stiffness, employing metrics: the augmentation index, pulse-wave velocity, and carotid intima-media thickness. Furthermore, the bibliographies of the retrieved articles were examined to identify the validated literature.

Given the innovative approach to the topic, the inclusion criteria were intentionally broadened to maximize the scope of the study concerning ED among PsA patients. However, several potential confounding factors were assessed, including comorbidities in PsA patients, medication intake, the absence of a specified inflammatory joint disease, or the simultaneous assessment of several joint diseases in one study group without subgroup analysis. Additionally, significant limitations of the studies were reviewed, such as the failure to distinguish PsA patients from those with psoriasis, treating them as a single group, and the lack of information regarding the patients' current treatment stage (whether the disease is controlled or newly diagnosed, etc.). Comparative indicators for RA were chosen for comparative purposes without special selection criteria, beyond assessing the study's internal validity.

Given the limited availability of specific data, we used the findings on the status of ED in RA as a benchmark for comparative analysis, provided that these findings were not affected by publication bias. This approach was aimed at strengthening the validity of our results [10].

## Endothelial dysfunction in PsA

### Coronary flow reserve

Coronary flow reserve (CFR) is a highly sensitive marker that can predict the occurrence of severe coronary stenosis and is a diagnostic marker of coronary artery disease (CAD) [5]. The work by Atzeni et al. highlighted a notable reduction in CFR among patients with PsA (2.86 ± 0.70 compared to a normal range of 3.3 ± 0.43; *p* < 0.01) [11]. This finding is consistent with the related work that demonstrated a significant reduction in CFR in patients with PsA compared to healthy individuals (1.9 ± 0.3 vs 3.6 ± 0.2; *p* < 0.002) [12]. Retrospective analysis research carried out in patients with PsO among whom there were also individuals with PsA further corroborated these findings. The study identified coronary microvascular dysfunction, defined as CFR < 2.5, and found that it is associated with more severe PsO (OR = 3.1; *p* = 0.03) and the presence of PsA (OR = 2.9; *p* = 0.03) [13]. These observations are supported by other researchers [14–16]. Furthermore, elevated levels of asymmetric dimethylarginine (ADMA) in patients with PsA have been found to correlate with significantly reduced CFR, indicating ED in central microcirculation, similar to the findings in patients with RA. In a study by Puig et al., the impact of tumor necrosis factor inhibitor (TNFi) therapy on CFR was investigated. In patients with PsO but with no PsA without cardiovascular disease, CFR measurements in the left anterior descending coronary artery showed a significant increase after an average of 6 months of TNFi treatment, compared with baseline values [17]. This improvement in CFR was associated with reduced levels of TNF and high-sensitivity C-reactive protein (CRP), although it did not correspond to changes in the Psoriasis Area and Severity Index (PASI) [17]. This points to a discrepancy between the severity of ED and skin involvement (Table 1).

**Table 1:** Table summarizing key studies cited

Study [Ref]	Design	PsA (F)	Contr (F)	Findings	Conclusions
Atzeni et al. 2011 [11]	CFR measured via dipyridamole transthoracic stress echocardiography, ADMA levels from blood samples	22 (10) 54.9 ± 12.97	35 (16) 55.36 ± 12.97	Plasma ADMA levels were significantly higher in PsA patients (0.71 ± 0.07 µmol/l) compared to controls (0.48 ± 0.07 µmol/l; *p* = 0.00) CFR was significantly reduced in PsA patients (2.86 ± 0.70) vs controls (3.3 ± 0.43; *p* < 0.01) Significant correlation between CFR and plasma ADMA levels in PsA group (R = 0.28; *p* < 0.01)	PsA patients exhibited subclinical atherosclerosis with higher ADMA levels indicating endothelial dysfunction. ADMA is a potential marker for assessing endothelial dysfunction in PsA patients
Mahfouz et al. 2014 [12]	Impact of Neutrophil–Lymphocyte Ratio (NLR) on CFR and myocardial function PsA patients without coronary artery disease risk factors	68 (22) 42.5 ± 12.8	62 (17) 43.0 ± 13.2	NLR was significantly higher in PsA patients (7.86 ± 0.6) versus controls (1.39 ± 0.6; *p* < 0.0001) CFR was significantly lower in PsA patients (1.9 ± 0.3) compared to controls (3.6 ± 0.2; *p* < 0.002) Myocardial function was significantly impaired in PsA patients NLR and C-reactive protein levels were independent predictors of impaired CFR.NLR ≥ 3.6 were the cutoff for predicting impaired CFR	CFR is significantly reduced in PsA patients. High NLR is an independent and powerful predictor of impaired CFR and early myocardial dysfunction in PsA patients
Ikonomidis et al. 2022 [14]	Assessments included PBR using Sidestream Dark Field imaging, CFR, FMD of the brachial artery	126 PsA patients	185 psoriasis patients, 150 healthy controls	Patients with psoriatic disease has reduced CFR compared with controls (2.86 ± 0.93 versus 3.39 ± 0.68, *p* < 0.001) Patients with psoriatic disease had higher PBR but lower FMD, and GLS compared with controls Patients with psoriatic arthritis had more impaired CFR (2.75 ± 0.85 versus 2.96 ± 0.99, *p* = 0.045) and FMD (5.45 ± 3.2 versus 7.76 ± 4, *p* = 0.003) compared to those with plaque psoriasis	Psoriatic disease is associated with significant impairments in endothelial, vascular, and myocardial function. Coronary microcirculatory function and flow-mediated dilation are further compromised in psoriatic arthritis compared to plaque psoriasis
Yilmazer et al. 2015 [19]	Comparison of endothelial function and cIMT, assessed through FMD and NID % using brachial artery ultrasonography	20 (15) 41.4 ± 11.5	20 (11) 40.3 ± 8	FMD % was significantly lower in PsA patients compared to controls, indicating endothelial dysfunction FMD % did not significantly correlate with clinical and laboratory data of PsA patients	PsA patients without any CAD or traditional cardiovascular risk factors may have ED, supporting the potential link between PsA and atherosclerotic disorders. The primary indicator of subclinical atherosclerosis in this population is reduced FMD %
Gonzalez -Juanatey et al. 2007 [20]	Endothelial function assessed by measuring FMD% and endothelial independent vasodilation (GTN%)	50 (23) 49.7 ± 12.8	50 (23) 49.9 ± 12.7	FMD% was significantly lower (6.3% vs. 8.2%; *p* = 0.008) in PsA patients compared to controls, indicating endothelial dysfunction A significant correlation was found between C-reactive protein level and erythrocyte sedimentation rate at the time of disease diagnosis and FMD% (*p* < 0.04)	PsA patients without overt cardiovascular disease or classic cardiovascular risk factors exhibit endothelial dysfunction, as evidenced by impaired FMD% The study underscoring the importance of assessing cardiovascular risks regardless of the presence of traditional risk factors
Bilgen et al. 2018 [21]	Parallel group study on subclinical atherosclerosis through FMD and cIMT	30 (24) 43.8 ± 11.6	30 (24) RA patients, 44.1 ± 13.7 30 (24) healthy controls 46.1 ± 12.2	FMD was significantly lower in both PsA and RA patients compared to healthy controls, indicating impaired endothelial functions Median CIMT was higher in RA patients than in PsA patients and healthy controls (*p* = 0.008), suggesting greater atherosclerosis presence	Both PsA and RA patients exhibit impaired endothelial functions in the absence of conventional cardiovascular risk factors or overt cardiovascular disease, indicating a potential link between these inflammatory musculoskeletal diseases, atherosclerosis, and cardiovascular disease
Turonova et al. 2018 [22]	ED in children with juvenile psoriatic arthritis (JPSA), vascular measurements conducted for FMD%	25 (14) 13.91 ± 2.98	25 (14) 14.01 ± 3.0	JPSA patients had significantly lower FMD% compared to healthy controls. Strong correlations observed between lower FMD% and longer disease duration, higher levels of non-specific inflammatory markers, and greater functional disability	Endothelial dysfunction is present in Slovak children with JPSA even without classic cardiovascular risk factors, and its severity is associated with early disease onset, increased disease activity, and disability
Sharma et al. 2014 [23]	Comparative study involving FMD% and NMD% evaluation	40 (17) 42 ± 9.57	40 (13) 39 ± 4.3	Significant impairment in FMD% was observed in PsA patients compared to controls, indicating endothelial dysfunction. No significant difference in NMD% between patients and controls	Endothelial dysfunction is present in PsA patients independent of traditional cardiovascular disease risk factors, highlighting the intrinsic association between PsA and cardiovascular morbidity
Shang et al. 2012 [26]	Assessment of ventricular and arterial stiffness	43 (20) PsA patients without HT or LVH	30 (16) PsA patients with HT or LVH, 50 (22) healthy Controls	Significantly increased ventricular and arterial stiffness in PsA patients (*p* < 0.001), including those without hypertension/LV hypertrophy	PsA patients exhibit increased ventricular and arterial stiffness irrespective of LV remodeling evidence, with long-standing disease duration posing a higher risk. The inflammatory burden of PsA could be a key contributor to early cardiovascular disease development
Patschan et al. 2018 [27]	Comparison of eEPC system with cardiovascular risk factors and vascular stiffness parameters	31 (15) 47.7 ± 2.0	26 (N.D.) healthy subjects 30 (13) PSO patients 49.0 ± 2.8	No correlation between vascular stiffness parameters (PWV and AIX) and eEPC colony formation or circulating numbers Disease duration, severity, individual pain, CRP values, and history of biological treatment did not significantly affect the eEPC system or vascular stiffness parameters	Vascular stiffness, as measured by PWV and AIX, does not significantly differ in patients with Ps/PsA compared to healthy subjects, suggesting that pulse-wave analysis may not be a suitable method for assessing cardiovascular risk in autoimmune-mediated diseases like Ps and PsA
Triantafyllias et al. 2022 [33]	Assessment of aortic stiffness and CV risk using cfPWV	112 (62) 55 years	88 (71) 51 years	cfPWV was significantly higher in PsA patients compared to healthy controls (*p* < 0.001), even after adjusting for confounding factors	PsA patients exhibit higher aortic stiffness and, consequently, an increased CVR compared to healthy individuals. cfPWV, which correlates with MAP and the ESC-SCORE, emerges as a valuable tool for identifying high-risk PsA patients
Costa et al. 2012 [34]	Assessment of arterial stiffness through aortic PWV measurement using a SphygmoCor device	20 (6) 38.6 years	20 (6) 38.7 years	Significantly higher aPWV in PsA patients compared to controls, even after adjusting for age, weight, height, heart rate, and central mean pressure (PsA: 8.3 ± 0.2 m/s vs. control: 6.8 ± 0.2 m/s; *p* < 0.0001) Among PsA patients, aPWV was correlated with the known duration of disease (*r* = 0.63; *p* = 0.003), a relationship that persisted after adjusting for main confounders (*β* = 0.011; *p* = 0.013)	Psoriatic disease, including PsA, is a systemic condition that affects arterial vessels, indicating the presence of pathogenetic mechanisms that may accelerate the atherosclerotic process. PsA patients exhibit increased arterial stiffness, suggesting an elevated cardiovascular risk associated with disease duration
Kimhi et al. 2007 [41]	Assessment of Subclinical atherosclerosis by measuring the IMT of the common carotid artery. Carotid duplex scanning assessed IMT, while traditional CVR factors were also evaluated	47 (24) 50 ± 13.5	100 (51) 34.9 ± 11.9	PsA patients had significantly higher average IMT compared to controls (0.76 ± 0.11 vs. 0.64 ± 0.27; *p* < 0.00001), even after adjusting for age, gender, BMI, hypertension, and hyperlipidemia. PsA patients showed higher levels of hypertension, hyperlipidemia, ESR, CRP, and fibrinogen	PsA patients exhibit greater IMT, indicating higher subclinical atherosclerosis, which correlates independently with disease activity and traditional atherosclerosis risk factors. This underscores the need for CVR assessment and management in PsA patients
Tam et al. 2008 [42]	Assessment of the prevalence of subclinical atherosclerosis in PsA patients compared to age, sex, and ethnicity-matched healthy controls, using carotid USG to measure IMT at various carotid artery sites	82 (40) 49 ± 10	82 (40) 50 ± 10	After adjusting for traditional CVR factors, PsA patients exhibited a higher prevalence of subclinical atherosclerosis. Increased sugar and total triglyceride levels were independent explanatory variables for subclinical atherosclerosis in PsA	PsA is associated with an increased prevalence of subclinical atherosclerosis. Carotid IMT is a useful tool for identifying PsA patients with subclinical atherosclerosis, suggesting the need for early intervention in these patients
Garg et al. 2016 [43]	Cross-sectional study comparing PsA patients with age and sex-matched controls. It assessed CIMT as a marker of atherosclerosis, along with FMD for endothelial function, and various inflammation markers cytokines, and ED indicators	18 (7) 43.81 ± 8.33	18 (8) 42.93 ± 10.16	CIMT was significantly higher in PsA patients compared to controls (0.062 ± 0.18 vs. 0.045 ± 0.10 cm, *p* < 0.01). FMD percentage, EPCs percentage, and HDL cholesterol were significantly reduced in PsA patients compared to controls. PsA patients showed significantly increased levels of ESR, CRP, TNF-α, IL-6, ICAM-1, and VCAM-1	PsA is associated with endothelial dysfunction and accelerated atherosclerosis, as indicated by impaired FMD and increased CIMT. Inflammatory mechanisms related to PsA, including TNF-α and IL-6, and markers of vascular dysfunction are implicated in the development of vascular disease, suggesting cytokine-driven inflammation contributes to ED and atherosclerosis in PsA
Eder et al. 2008 [44]	Case control study investigating the prevalence of subclinical atherosclerosis in PsA patients compared to age, sex, and atherosclerotic risk factor-matched controls. It involved a duplex scan of the carotid arteries to evaluate carotid cIMT and record the presence and grading of atherosclerotic plaques to calculate the carotid plaque index	40 (28) 57.85 years	40 (28) 57.05 years	PsA patients exhibited a higher IMT (1.04 ± 0.35 mm) compared to controls (0.88 ± 0.29 mm; *p* = 0.03) PsA patients also had a higher carotid plaque index (2.3 ± 2.6) than controls (1.12 ± 2.09; *p* = 0.03) Multivariate analysis showed PsA status, age, and triglyceride levels were associated with the presence of carotid plaque Traditional risk factors were more prevalent in PsA patients but did not reach statistical significance	PsA patients may have an increased prevalence of subclinical atherosclerosis, not fully explained by traditional risk factors. This underscores the need for vigilant monitoring and strict control of atherosclerotic risk factors in PsA patients
Lam et al. 2020 [46]	Cohort analysis of Psoriatic Arthritis (PsA) patients followed since 2006 to investigate the potential of Disease Activity in Psoriatic Arthritis (DAPSA) to predict cardiovascular (CV) events, independent of traditional CV risk factors, carotid plaque (CP), and carotid intima-media thickness (CIMT)	189 (85) 48.9 ± 11.6	Control: 154 (64) undergoing carotid ultrasound assessment 49.4 ± 11.6	Higher DAPSA was significantly associated with an increased risk of CV events (HR: 1.04, 95% CI (1.01–1.08), *p* = 0.009), even after adjusting for all CV risk scores Carotid plaques were found in about 20% of the patients In the subgroup with carotid ultrasound, 23 (14.9%) experienced a CV event. CP presence was associated with an increased risk of CV events, with HRs ranging from 2.35 to 3.42 after adjusting for CV risk scores and DAPSA	Higher DAPSA levels and the presence of CP are independent predictors of CVD events in PsA patients, suggesting the need for integrating inflammatory disease activity and subclinical atherosclerosis measures in CVR assessment and management strategies for PsA patients
Lucke et al. 2016 [47]	Screening for carotid plaque at baseline and follow-up with duplex ultrasound to identify the frequency of carotid plaque in asymptomatic PsA patients at baseline and follow-up screening, and assess the impact of demonstrating plaque on management of traditional cardiovascular risk factors	87 (46)	No control	Carotid plaque was present in 34 out of 87 (39%) PsA patients. Age and triglyceride levels were predictors of plaque presence. Patients with plaque had a tendency towards higher rates of smoking, diabetes, and higher LDL levels. The use of biologic medications for PsA was high (75%), compared to similar patient cohorts with carotid plaque. No correlation was found between disease duration or activity and the presence of carotid plaque	Despite the high cardiac risk indicated by the presence of carotid plaque, there is a gap in the implementation of preventive cardiovascular measures in PsA patients, highlighting the need for improved cardiovascular risk management in this group
Cheng et al. 2020 [48]	The study evaluated the utility of carotid ultrasound (US) parameters (carotid intima-media thickness [cIMT], the presence of plaque, total plaque area [TPA]) alone or combined with the Framingham Risk Score (FRS) in distinguishing PsA patients with and without CAD, using high-resolution US and coronary CT angiography (CCTA) in 91 PsA patients without overt cardiovascular diseases	91 (35) 50 ± 11	No control	Thirty-five (38%) patients had carotid plaque Fifty-four (59%) patients exhibited CAD (CAD+), and 9 (10%) had obstructive-CAD (O-CAD+) No significant association was found between the presence of carotid plaque and CAD Both cIMT and TPA were higher in CAD+ and O-CAD+ groups compared to their negatives Mean cIMT was an independent predictor for CAD and O-CAD, while maximum cIMT and TPA were independent predictors for O-CAD after adjusting for covariates.	US parameters including cIMT and TPA, in addition to FRS, may enhance cardiovascular risk stratification in PsA patients, offering valuable insights for better identifying those at higher risk for CAD and O-CAD
Szentpetery et al. 2013 [51]	The study investigated the impact of metabolic syndrome and psoriatic disease-related variables on coronary plaque burden in PsA patients without symptoms of CAD, comparing 25 with metabolic syndrome to 25 without, and to 50 age- and sex-matched controls. Participants underwent 64-slice coronary computed tomography angiography to evaluate plaque localization, segment involvement score (SIS), segment stenosis score (SSS), and total plaque volume (TPV), with plaques classified as calcified, mixed, or noncalcified	50 PsA patients (25 with metabolic syndrome, 25 without)	50	76% of PsA patients had plaques compared to 44% of controls (*p* = 0.001) PsA patients had higher SIS, SSS, and TPV than controls (*p* = 0.003, *p* = 0.001, and *p* ≤ 0.001, respectively). Mixed plaques were more prevalent in PsA patients, with a higher volume compared to controls (*p* < 0.001). No significant difference in plaque burden or type was observed between PsA patients with and without metabolic syndrome TPV was associated with PsA diagnosis but not with metabolic syndrome. Age, highest CRP level, highest swollen joint count, disease duration, and plasma glucose level were independent predictors of higher plaque burden in PsA patients	Psoriatic arthritis is linked to increased coronary plaque formation, especially mixed plaques, regardless of metabolic syndrome presence. Disease activity and severity in PsA may better predict coronary plaque burden than traditional cardiovascular risk factors

Below the number of studied patients there is mean (SD) age of the subjects

*PsA* psoriatic arthritis, (*F*) number of female participants

### Flow-mediated dilatation

Flow-mediated dilation (FMD) of the brachial artery is a reliable method for assessing nitric oxide (NO) dependent endothelial function in conduit arteries and early stages of atherosclerosis [6, 18]. Numerous studies have consistently shown that FMD is significantly impaired in patients with PsA compared to healthy individuals. This reduction in FMD has been observed even in patients without traditional CVRF, including in cases of juvenile PsA [19–22]. These findings support the idea of a higher incidence of macrovascular ED in PsA, mainly in the form of impaired NO release, as suggested by preserved nitroglycerin-mediated dilatation (NMD). Correction of a decrease in FMD [%] after sublingual nitroglycerin administration was observed in two independent studies [20, 23]. These results showed a strong correlation with CRP levels, a known factor in promoting ASCVD in part through the downregulation of endothelial nitric oxide synthase transcription [24]. The other important factor that contributes to the impaired NO release may be elevated levels of ADMA, an endogenous inhibitor of endothelial nitric oxide synthase, which has been found to be increased in ASCVD [25] and in patients with PsA [11]. It is considered a possible marker of subclinical ASCVD, however, its levels were noted to decrease in RA patients after treatment. This finding suggests that ADMA levels may not be elevated in patients with PsA treated with disease-modifying anti-rheumatic drugs (DMARDs) or biologic therapies [11, 19].

## Arterial stiffness in PsA

### Augmentation index (AIx)

**Fig. 1:**
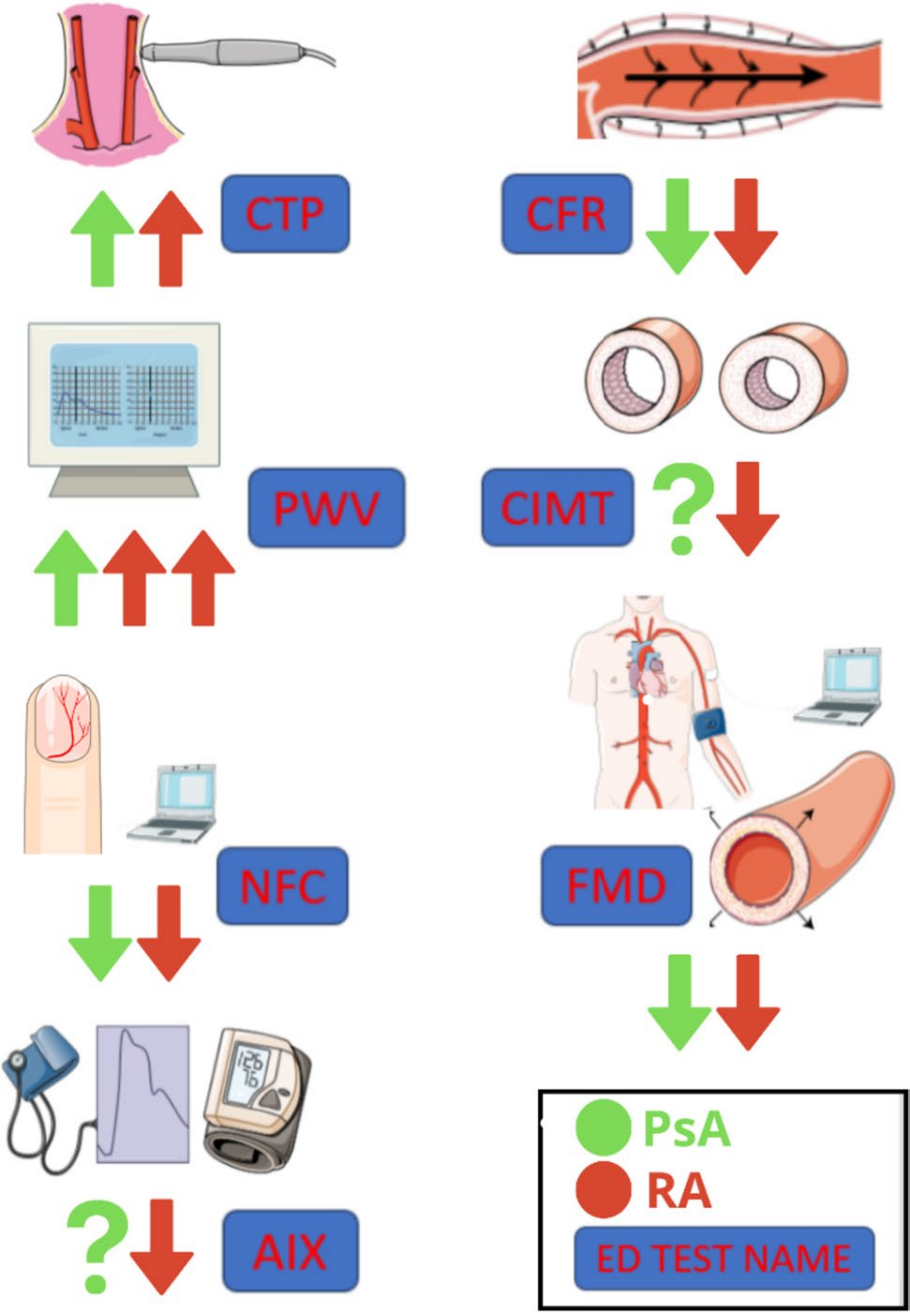
A Comparative analysis of endothelial dysfunction (ED) and subclinical atherosclerosis in psoriatic arthritis (PsA) and rheumatoid arthritis (RA). This figure visualizes the outcomes of endothelial dysfunction and subclinical atherosclerosis tests, comparing PsA with RA. Each disease is distinguished by specific colors for easy identification. An upward arrow indicates results higher than those of healthy controls, signifying elevated levels, while a downward arrow indicates reduced levels. A question mark denotes inconclusive outcomes. The test names are clearly labeled within frames for quick reference. *CTP* carotid total plaque, *CFR* coronary flow reserve, *PWV* pulse wave velocity, *CIMT* carotid intima media thickness, *NFC* nailfold capillaroscopy, *FMD* flow-mediated dilatation, *Aix* Augmentation Index Creation Details: The figure was designed using Microsoft PowerPoint and Canva (https://www.canva.com/pl_pl/) on February 3, 2024. Online access at https://www.canva.com/design/DAF7vdCJ41s/aCvx1TqqhiFH-V0dLY-lVw/view. Certain illustrations within the figure are adapted from Servier Medical Art (https://smart.servier.com), courtesy of Servier, under a Creative Commons Attribution 3.0 Unported License available at https://creativecommons.org/licenses/by/3.0/

The augmentation index (AIx) is a marker of arterial stiffness. Its increase is one of the first signs of vascular dysfunction. Shang et al. showed that individuals with PsA have higher AIx values as compared to healthy controls [26], however, those results have not been replicated in other studies. In addition, conflicting data have been obtained regarding the association between disease activity and AIx. In research by Angel et al., a mixed group of patients with RA, PsA, and ankylosing spondylitis was evaluated. It was revealed that fluctuations in disease activity, associated with cyclical character of long-term infliximab treatment, were not accompanied by significant changes in arterial stiffness indices, including AIx [28]. However, two studies that evaluated the importance of the treat-to-target approach solely in PsA, showed that achieving a sustained minimal disease activity (sMDA) and sustained low disease activity, as measured by the PsA Disease Activity Score (PASDAS), resulted in reduction of AIx [29, 30]. Therefore, due to inconsistent results, drawing strong conclusions about the relationship between PsA and AIx is challenging (Fig. 1).

### Pulse wave velocity

Pulse wave velocity (PWV) is a measure of arterial diameter, pulse pressure, and pulsatile diameter change, which makes it an indicator of arterial distensibility. The aortic PWV is calculated as the difference in the travel time of the pulse waves between two different recording sites and the heart, divided by the travel distance covered by the pulse wave [31]. PWV was found to be elevated in patients with PsA compared to controls [32, 33], even in the early stages of the disease, and this increase was even more pronounced in those with long-term disease (8.2 ± 0.8 vs 6.8 ± 1.0 m/s, *p* < 0.001) [34]. The burden of inflammation over time was also revealed since PWV was related to erythrocyte sedimentation rate (ESR) and CRP levels, as well as DAS28 and the Bath Ankylosing Spondylitis Disease Activity Index (BASDAI) [35]. A higher PWV was associated with increased arterial stiffness and higher CV risk [36]. It was significantly related to a higher systolic and diastolic blood pressure, as well as a higher body mass index, all of which are possible causes of reduced arterial distensibility prevalent in patients with PsA [32, 37]. Furthermore, a different study suggested that TNFis may improve arterial stiffness as measured by PWV. However, the use of a mixed cohort of patients with RA, AS, and PsA makes it challenging to draw disease-specific conclusions [38].

## Vascular remodeling in PsA

### Carotid intima-media thickness

Carotid intima-media thickness (cIMT) is considered a sensitive marker of subclinical ASCVD that can be measured using a non-invasive B-mode ultrasound [39, 40]. It has been evaluated in patients with PsA with mixed results. Several studies found significantly increased cIMT both in patients with and without CVRF [41–44]. Smaller but still significant increases were observed in the general population of PsO, who showed higher cIMT than a subgroup with PsA [45]. Elevated cIMT is an independent risk factor aggravated by the coexistence of common comorbidities such as older age, body mass index, waist-to-hip ratio, systolic and diastolic blood pressure, and the presence of diabetes. All were positively correlated with cIMT, along with some disease-related parameters such as duration of disease and activity (ESR, CRP), age of onset, duration of skin and joint manifestations. Although in some studies cIMT levels were not different between the PsA and control groups [11, 19], it is speculated that this difference stemmed from the increased use of anti-TNF treatment. To our knowledge, there is still no targeted research to support this claim [21].

### Carotid plaques

One of the most important indicators of subclinical ASCVD is the burden of carotid plaques. It can be measured using a carotid duplex ultrasound and is defined as a localized thickening of the arterial wall [46]. Patients with PsA have a higher prevalence of carotid plaques than the general population [44], especially in older people or patients with a history of smoking, hypertension, high levels of triglycerides, and high levels of low-density lipoprotein cholesterol. Studies have also shown a possible risk of rapid progression from unilateral to bilateral plaques and the development of new plaques in less than 2 years from the initial screening among patients with CVRF [47]. Thus, carotid plaque burden (CPB) can be a predictor of future CV events in PsA patients with subclinical atherosclerosis [46, 48]. It is independent of Disease Activity in Psoriasis (DAPSA) and traditional CVRF, and is associated with a threefold increase in the risk of developing CV events in the PsA population [46]. Patients with plaques have a longer duration of the disease and more swollen joints, although they share a similar CV risk profile with the plaque-free group [49]. Carotid total plaque area (CTPA) shows the sum of the extent of the carotid plaques and is likely a better surrogate marker than carotid intima-media thickness (cIMT) alone. Interestingly, it was found to be higher in patients with PsA compared to patients with PsO without arthritis. It was associated with an increase in the intensity of inflammation and in the duration of PsA [50]. DAPSA was also found to be an independent predictor of CV events with better sensitivity than a tender or swollen joint count [46]. In addition to carotid plaques, the burden of coronary plaques and the prevalence of CAD in PsA should also be considered. It is measured using coronary computed tomography angiography (CCTA), a non-invasive method comparable to invasive angiography that offers a quantitative and qualitative evaluation of stenotic and non-stenotic coronary plaque burden (coPB) with high precision [51, 52]. Coronary plaques are considered to be at higher risk when they are noncalcified or mixed, while calcified plaques (CP) are deemed less vulnerable. All are prevalent in PsA even when they do not cause vessel obstruction or symptomatic disease [53]. Shen et al. found in their study that 60% of patients with PsA had at least one type of plaque. Compared to controls, they had a two to threefold increase in prevalence of all types of plaques, particularly mixed plaques/noncalcified plaques (MP/NCP), which were associated with longer disease duration. Each additional year of exposure to inflammation was estimated to increase the risk of developing MP/NCP by 6%. Patients with PsA were also more likely to have three-vessel disease, obstructive lesions, and a higher segment involvement score (SIS) than controls, all indicating a higher coPB [54]. It is believed that SIS has the capability to predict major adverse cardiac events in asymptomatic patients with an initially higher risk of CAD [51]. No association was demonstrated between carotid and coronary plaque; however, mean and maximum cIMT were significantly associated with the latter [54]. In a related study, the same finding was reported, indicating that although CPB may not be a precise marker of CAD, an increase in mean cIMT was an independent explanatory variable associated with the disease [48]. These data suggest that carotid ultrasound evaluation may be a reasonable tool for CV risk assessment in asymptomatic patients, certainly better than traditional risk scores, however, not as good as CCTA [55]. A major advantage of CCTA is that it can detect the non-stenotic burden of ASCVD, which is present in patients well before the diagnosis of CAD. A study by Szentepery et al. compared patients with PsA but without CAD symptoms with matched controls. They found that PsA was associated with a higher number and extensiveness of coronary plaques, particularly of mixed type, however, there was no difference in CP and NCP. Interestingly, plaques were predominantly located in LAD coronary segments, which are generally associated with a worse prognosis. Furthermore, lipid-rich MPs are more at risk than CP, they are more often associated with thin-cap fibroatheromas, making them more prone to rupture. They are more prevalent in acute CAD compared to chronic disease. This might explain the relationship between PsA and cardiac events. To further strengthen this link, the SIS results, the segment stenosis score (SSS), and the total plaque volume (TPV) were higher in the PsA group and were correlated with measures of disease activity such as the maximum swollen joint count, maximum ESR rate, and CRP levels. Furthermore, age, disease duration, and plasma glucose level were independent predictors of a higher plaque burden in PsA. This suggests that minimizing disease activity, but only combined with optimal metabolic control, would be beneficial in preventing CV events. However, in this study, CAD was independent of the presence of metabolic syndrome in PsA. CoPB was not significantly higher in patients with characteristics of metabolic syndrome, as there was no difference in SIS, SSS, TPV, and type of plaque. TPV was associated with a diagnosis of PsA, but not with metabolic syndrome [51].

### Comparison to RA

RA is a multifactorial autoimmune disease that primarily affects the synovial joints, but also the extraarticular organs. Although both RA and PsA share synovitis as their hallmark feature, they vary in clinical presentation and the details of treatment.

### Endothelial dysfunction in microcirculation in RA

A meta-analysis performed by Erre et al. reported that CFR is significantly lower in patients with rheumatic diseases in general, suggesting analogous results between PsA and RA [56]. A study of patients with early RA that included 25 individuals with an average duration of 6.24 months revealed a decrease in CFR compared to healthy controls. It depicts a dysfunction of the coronary microcirculation even in the early stages of the disease [57]. CFR has also been found to be reduced in established RA in several studies [58–61]. Moreover, it was observed that CFR was inversely correlated with disease activity as measured by DAS 28, disease duration, and CRP [61]. Another study showed that coronary microvascular dysfunction, defined as CFR < 2, was associated with an increased risk of all-cause mortality in patients with RA and diabetes. Patients with coronary microvascular dysfunction were also more likely to die from cardiovascular disease. A significant decrease in CFR is similar to that in patients with PsA [60]. In a study involving nailfold capillaroscopy (NFC), a decreased capillary density was observed in patients with RA compared to the control group. The study revealed a negative correlation with CRP and PWV, while showing a positive correlation with HDL-C and cardiac index. Importantly, capillary density was also significantly associated with CVR calculated using the Framingham Risk Score (FRS). This implies that NFC could act as an important tool for estimating CVR in RA patients [62]. No similar associations were found with the NFC findings in PsA. Abnormal findings in NFC are common in patients with PsA and RA and both these groups are characterized by similar morphological abnormalities, including tortuosity and reduced capillary density, but, in contrast to RA, their significance for overall ED and CVR in PsA remains unknown.

### Endothelial dysfunction in macrocirculation in RA

FMD has been widely investigated in patients with RA and its impairment has been observed in numerous studies [63]. One reported that even people with early RA have altered FMD when compared to controls, suggesting that ED can be detected regardless of the short course of the disease [64]. In terms of factors that may be associated with ED measured by this parameter, it was revealed that lower values of FMD [%] were correlated with certain shared epitope alleles: HLA-DRB1*04 and HLA-DRB1*0404. Furthermore, the same research evaluated endothelium-independent vasodilation after nitroglycerin administration, which was not significantly different between the study and the control group, further implicating impaired NO release as a major contributor to the vascular alterations present in patients with RA [65]. Other researchers found an association between decreased FMD and CRP levels, suggesting that inflammation plays an important role in people with ED in RA [66, 67]. Additionally, one of these studies found an association between FMD and CRP [67]. Interestingly, CCR5Δ32 deletion appears to be a protective factor for ED measured by FMD. The CCR5 receptor is present in T lymphocytes and antigen-presenting cells, including macrophages or dendritic cells, and is involved in trafficking and activation [68, 69]. Notably, it is also expressed in vascular smooth muscle cells [70]. Rodrguez-Rodrguez et al. reported in their work that patients with RA who are carriers of this deletion presented significantly higher FMD values compared to other patients, implying that the CCR5 molecule may play a role in the development of ASCVD in RA [71].

### Arterial stiffness in RA

A study by Becetti et al. showed that patients with RA had elevated AIx compared to controls. These values were associated with albuminuria, as assessed by the urinary albumin-to-creatinine ratio, which was also directly correlated with vascular cell adhesion molecule 1 (VCAM-1) levels, an indicator of endothelial activation, and inversely with interleukin-10 levels, an anti-inflammatory cytokine. This suggests that not only hypertension or diabetes but also albuminuria could be another marker of systemic vascular damage in RA [72]. Noteworthy data depicted a direct correlation between aortic PWV and epicardial adipose tissue in patients with RA [73]. Epicardial adipose tissue is a layer of fat located between the surface of the heart and the visceral pericardium [74]. Physiologically, it balances the amount of free fatty acids within the myocardium, plays a role in thermoregulation, and mechanically protects the autonomic nerves and ganglia that innervate the heart muscle [75]. However, in pathological conditions such as obesity, due to its proximity to the myocardium and its shared microcirculation with this tissue, it is believed to play a specific role in the pathogenesis of CAD through paracrine and so-called vasocrine activity, via the coronary vasa vasorum. It releases numerous factors, such as pro-inflammatory cytokines, pro-fibrotic agents, or metalloproteinases, which contribute to the progression of CV disease [76, 77]. Additionally, increased arterial stiffness has been found to alter left ventricular function. One study found an association between elevated PWV and increased filling pressure of the left ventricle [78], while another study showed that the left ventricular myocardial performance index, an indicator of systolic and diastolic functions, was positively correlated with carotid-femoral PWV and AIx corrected for heart rate [79]. These studies suggest that aortic stiffness could act as an indicator of not only vascular damage but also heart muscle involvement, thereby increasing the CVR in RA patients. A study that evaluated PWV in patients with early RA and PsA revealed that both groups had a higher PWV compared to controls, but it was significantly higher in individuals with RA (PsA vs RA vs HC, mean ± SD, 6.42 ± 1.39 vs 7.91 ± 1.93 vs 5.11 ± 0.83 m/s, *p* < 0.001; PsA vs RA, *p* = 0.009) [80]. This suggests that arterial stiffness increases in the early stages of both types of arthritis, but it seems to be more pronounced in RA.

### Vascular remodeling

Increased values of cIMT have been frequently observed among patients with RA compared to controls [81, 82]. Some studies have found associations between this marker and indices of inflammation or disease activity, such as CRP [83–85] and DAS28 [86, 87]. In a study conducted in patients with early RA, cIMT was increased but still within the normal range compared to healthy controls [57], while the other research showed an increase in cIMT in early RA. However, it revealed that this marker did not differ significantly between early PsA patients and healthy controls [80]. These results suggest that vascular remodeling is present in RA even in the early stages of the disease, whereas in PsA it is less apparent. Patients with RA had significantly increased cIMT, which was also positively associated with Homeostatic Model Assessment for Insulin Resistance (HOMA2-IR). It indicates that impaired insulin sensitivity could be another potential risk factor for the development of ASCVD in patients with RA [86]. Another study found that an increase in cIMT in RA was correlated with higher levels of highly pro-inflammatory CD4+/CD28+ T lymphocytes and higher expression of the fractalkine receptor (CX3CR1) on them, which is responsible for interaction with the chemokine CX3CL1 [87]. Increased numbers of these cells have been observed among patients with unstable angina compared to patients with stable angina, suggesting their role in the infiltration of unstable plaques [88, 89]. In addition to its role as a chemotactic factor, CX3CR1 can also act as an adhesion molecule for leukocytes, expressed in activated endothelial cells [90]. Therefore, upregulation of the fractalkine receptor may allow T cells to infiltrate the tissue and cause its damage, accelerating the atherosclerotic process [87]. In other relevant research FMD combined with cIMT was compared among individuals with RA, PsA, and controls. Patients with CV disease or pre-existing traditional CVRF were excluded from the investigation. In terms of FMD, the RA and PsA groups had lower values of this marker compared to controls. However, cIMT was significantly elevated only in the RA group, while the result in the PsA group was similar to healthy controls. Furthermore, carotid plaques, defined as thickening of intima-media > 1.0 mm, were detected only in the RA group, with no such cases in the PsA and control groups. The researchers suggested that the reason for this outcome might be the greater use of anti-TNF treatment in the PsA group, which has been shown to significantly reduce cIMT in psoriatic patients [91, 92], thus improving vascular remodeling in this group [21].

### Carotid plaques

Similarly to PsA, patients with RA are more susceptible to develop carotid plaques than the general population [93, 94]. Both CPB and cIMT are significantly associated with CVRF, consequently stroke and myocardial infarction, which can be avoided by identifying asymptomatic ASCVD in RA. [95] Evans et al. found an association between the presence of unilateral carotid plaque at baseline and an increased risk of acute coronary syndrome (ACS). The incidence of ACS could even increase fourfold if the plaques were present in both internal carotid arteries [96]. The impact of ASCVD on ACS in patients with RA may be an indication for the use of carotid ultrasound when evaluating CVR [97]. A study by Karpouzas et al. used computed tomography angiography to establish that noncalcified coronary plaque was found in 54% of afflicted arteries of asymptomatic RA patients compared to 21% in controls. Additionally, the plaque was more extensive in RA patients [98]. Furthermore, significantly elevated CRP levels have an impact on the appearance of unstable coronary plaque in CCTA [97, 99]. Increased CPB, CTPA, and coPB are associated with an elevated risk of CV events and ACS in patients with RA and, to a lesser extent, in PsA.

## Discussion

As observed in other studies, patients with PsO as well as patients with PsA have increased cardiovascular risk defined as a greater likelihood of major adverse cardiac events and a higher incidence of cardiometabolic comorbidities such as arterial hypertension, hypercholesterolemia, diabetes, obesity, metabolic syndrome, or non-alcoholic fatty liver disease compared to the general population. There is an unmet need for improvement in the primary or secondary prevention of cardiovascular diseases in patients with PsO and PsA by implementing the appropriate pharmaceutical interventions and adjusting risk stratification. Research shows that the use of traditional risk algorithms underestimates the likelihood of future CV events in patients with PsA [46, 48]. The widely used Framingham Risk Score (FRS) takes into account age, sex, smoking, hypertension, total cholesterol, and HDL-C but overlooks the inflammatory burden, diversity of PsA clinical phenotype, and a vast range of comorbidities. When comparing FRS and Systematic Coronary Risk Evaluation (SCORE) with cIMT and CTPA, an alarming number of patients must be reclassified into higher-risk groups based on ultrasound results. These imaging findings improve risk stratification, and the presence of carotid plaques is even more predictive than cIMT. The DAPSA score was associated with reclassification when using the SCORE algorithm, further demonstrating the influence of chronic inflammation and active disease [100, 101]. The European Alliance of Associations for Rheumatology (EULAR) recommends a multiplication factor of 1.5 for risk scores in RA patients only. Still, research suggests that similar considerations should apply to PsA [102]. This is particularly noteworthy because, upon comparing both groups, CTPA and the severity of subclinical ASCVD are even higher in PsA. These results are independent of traditional CVRF and show how suboptimal traditional scales are for stratifying CVR in PsA [50]. It has been shown that the progression of subclinical ASCVD can be slowed by achieving sMDA. There are two other measures of disease activity that should be considered in the context of reducing the general inflammatory burden. These are the DAPSA index, which includes the number of swollen and tender joints, CRP, visual analogue scale for pain as well as disease activity assessment, and PASDAS, which similarly to sMDA, includes peripheral joint, but also skin, and enthesitis domains. Sustained low disease activity according to PASDAS was shown to prevent carotid ASCVD and progression of arterial stiffness. The same was not achieved for DAPSA. This difference may suggest that some components, such as skin manifestation, may influence the severity of the disease more than previously anticipated [30]. Interestingly, a promising anti-TNF therapy failed to achieve the anti-atherogenic effect apart from the improvement in lipid profiles [103]. The role of this work was not to assess the benefits of anti-TNF therapy, although it is speculated that this treatment may have been, after all, beneficial in different ways and may have accounted for the variability in the study results, including studies comparing RA and PsA. Data suggest that indicators of arterial stiffness, including AIx and PWV, improve during TNFi treatment and the lower prevalence of vascular remodeling expressed as increased cIMT in PsA suggests the efficacy of this therapy.

## Conclusions

Evidence from the literature shows that PsA, similarly to RA, leads to ED over the course of the disease. ED plays an important role in the pathophysiology of cardiometabolic diseases. PsA has been found to be associated with the accelerated atherosclerosis process, which may also be related to the prevalence of metabolic syndrome. PsA and other forms of arthritis or autoimmune inflammatory diseases should be further investigated to determine the extent of their negative impact on the endothelium, and consequently the significance of their role in the development of ASCVD. Patients burdened with inflammatory diseases, especially arthritis, should receive special attention during the assessment of cardiovascular risk. Minimisation of PsA and RA activity, tight control of classic CVRF, as well as early detection of asymptomatic cases of ASCVD, could be beneficial in preventing CV events and limiting CV mortality. The EULAR recommendation to multiply CVR score by 1.5 in patients with RA should also be considered with respect to patients with PsA, whose CTPA and consequently the severity of subclinical ASCVD is even higher than in patients with RA. Finally, anti-TNF treatment appears to have a beneficial effect on endothelial function in patients with PsA by reducing markers of inflammation and cIMT, thereby improving vascular remodeling, which, in contrast, is more pronounced in patients with RA. However, further research is needed, as long-term reversal of ED and atherosclerosis progression is not easily attainable, even at the age of biologic and targeted synthetic drugs used in RA and PsA.

## Abbreviations

ACS: Acute coronary syndrome
ADMA: Asymmetric dimethylarginine
AIx: Augmentation index
ASCVD: Atherosclerotic cardiovascular disease
BASDAI: Bath Ankylosing Spondylitis Disease Activity Index
CAD: Coronary artery disease
CCR: CC chemokine receptor
CCTA: Coronary computed tomography angiography
CFR: Coronary flow reserve
cIMT: Carotid intima-media thickness
coPB: Coronary plaque burden
CP: Calcified plaques
CPB: Carotid plaque burden
CTPA: Carotid total plaque area
CV: Cardiovascular
CVR: Cardiovascular risk
CVRF: Cardiovascular risk factors
DAPSA: Disease Activity in Psoriatic Arthritis
DAS28: Disease Activity Score in 28 Joints
DMARDs: Disease-modifying anti-rheumatic drugs
EAT: Epicardial adipose tissue
ED: Endothelial dysfunction
ESR: Erythrocyte sedimentation rate
EULAR: European League Against Rheumatism
FMD: Flow-mediated dilatation
FRS: Framingham Risk Score
HDL-C: High-density lipoprotein cholesterol
HOMA-IR: Homeostatic model assessment for insulin resistance
hs-CRP: High-sensitivity C-reactive protein
IL: Interleukin
IR: Insulin resistance
MP: Mixed plaques
NCP: Noncalcified plaques
NFC: Nailfold capillaroscopy
NMD: Nitroglycerin-mediated dilation
NO: Nitric oxide
PASDAS: Psoriatic Arthritis Disease Activity Score
PASI: Psoriasis Area and Severity Index
PsA: Psoriatic arthritis
PsO: Psoriasis
PWV: Pulse-wave velocity
RA: Rheumatoid arthritis
SCORE: Systemic Coronary Risk Evaluation
SIS: Segment involvement score
sMDA: Sustained minimal disease activity
SSS: Segment stenosis score
TNF: Tumor necrosis factor
TNFi: Tumor necrosis factor inhibitor
TPV: Total plaque volume
